# Intrinsic disorder in the open reading frame 2 of hepatitis E virus: a protein with multiple functions beyond viral capsid

**DOI:** 10.1186/s43141-023-00477-x

**Published:** 2023-03-16

**Authors:** Zoya Shafat, Anwar Ahmed, Mohammad K. Parvez, Shama Parveen

**Affiliations:** 1 Centre for Interdisciplinary Research in Basic Sciences, Jamia Millia Islamia, New Delhi, India; 2 Centre of Excellence in Biotechnology Research, College of Science, King Saud University, Riyadh, Saudi Arabia; 3 Department of Pharmacognosy, College of Pharmacy, King Saud University, Riyadh, Saudi Arabia

**Keywords:** Hepatitis E virus (HEV), Open reading frame 2 (ORF2), Intrinsically disordered protein (IDP), Intrinsically disordered protein region (IDPR), Phosphorylation prediction, Molecular function

## Abstract

**Background:**

Hepatitis E virus (HEV) is the cause of a liver disease hepatitis E. The translation product of HEV ORF2 has recently been demonstrated as a protein involved in multiple functions besides performing its major role of a viral capsid. As intrinsically disordered regions (IDRs) are linked to various essential roles in the virus’s life cycle, we analyzed the disorder pattern distribution of the retrieved ORF2 protein sequences by employing different online predictors. Our findings might provide some clues on the disorder-based functions of ORF2 protein that possibly help us in understanding its behavior other than as a HEV capsid protein.

**Results:**

The modeled three dimensional (3D) structures of ORF2 showed the predominance of random coils or unstructured regions in addition to major secondary structure components (alpha helix and beta strand). After initial scrutinization, the predictors VLXT and VSL2 predicted ORF2 as a highly disordered protein while the predictors VL3 and DISOPRED3 predicted ORF2 as a moderately disordered protein, thus categorizing HEV-ORF2 into IDP (intrinsically disordered protein) or IDPR (intrinsically disordered protein region) respectively. Thus, our initial predicted disorderness in ORF2 protein 3D structures was in excellent agreement with their predicted disorder distribution patterns (evaluated through different predictors). The abundance of MoRFs (disorder-based protein binding sites) in ORF2 was observed that signified their interaction with binding partners which might further assist in viral infection. As IDPs/IDPRs are targets of regulation, we carried out the phosphorylation analysis to reveal the presence of post-translationally modified sites. Prevalence of several disordered-based phosphorylation sites further signified the involvement of ORF2 in diverse and significant biological processes. Furthermore, ORF2 structure-associated functions revealed its involvement in several crucial functions and biological processes like binding and catalytic activities.

**Conclusions:**

The results predicted ORF2 as a protein with multiple functions besides its role as a capsid protein. Moreover, the occurrence of IDPR/IDP in ORF2 protein suggests that its disordered region might serve as novel drug targets via functioning as potential interacting domains. Our data collectively might provide significant implication in HEV vaccine search as disorderness in viral proteins is related to mechanisms involved in immune evasion.

## Background

Hepatitis E virus (HEV) is a major zoonotic pathogen causing acute hepatitis E worldwide. HEV is a single-stranded RNA virus belonging to the family *Hepeviridae* [1]. According to recent data, about 15–110 million of the individuals worldwide are still experiencing infections and about 939 million of the world populations have already (past HEV) experienced infection from HEV [2]. In India, the reporting of hepatitis infections to the CBHI (Central Bureau of Health Intelligence) is exceptionally low, as mostly of the people share a common belief that the disease is characterized with lack of cure in allopathy, thus, establishing inaccurate burden of HEV infections. However as suggested, India has been reported with 10–40% cases of acute hepatitis and 15–45% cases of acute liver failure in its population [3, 4]. As reported in studies, the HEV is segregated into 8 genotypes (genotype 1–genotype 8). *Orthohepevirus A* species of the *Orthohepevirus* genus is segregated into 8 genotypes (genotype 1, genotype 2, GT genotype 3, genotype 4, genotype 5, genotype 6, genotype 7, and genotype 8). The genotypes 1 and 2 infect only humans and are major reasons for waterborne outbreaks in endemic regions of Africa, parts of Asia (South and Southeast) and Mexico while the genotypes 3 and 4 infect diverse mammals, such as humans, rabbits, swine and deer, and cause sporadic of hepatitis E cases in mainly developed countries of East Asia and Europe [5–9]. The genotypes 5 and 6 have been identified in Japan from wild boars [10, 11] while genotype 7 from dromedaries in Middle East countries and genotype 8 from China in Bactrian camels [12, 13]. The meat product (raw or undercooked) consumption from animals in developed nations is the chief cause of sporadic infections [14]. Moreover, transmissions such as blood-mediated [15], person-to-person [16], animal (pet) to human [17, 18] have been reported in patients, thus categorizing HEV as major concern of health issue. The diagnostic tests for HEV largely target mostly anti-HEV IgG and anti-HEV IgM antibodies. In diagnosis, initially HEV RNA and anti-HEV IgM antibodies are detected, which is followed up by anti-HEV IgG antibody detection. Presence or positive detection of anti-HEV IgM antibodies in serum are considered as important marker for acute HEV infection. Anti-HEV IgG antibodies have long-lasting persistence (duration is uncertain), thus are considered as markers for an individual who has experienced past infection [19, 20]. In contrast to this, anti-HEV IgM antibodies persist for a short duration of time (3 to 4 months), and thus are considered as markers in an individual who is undergoing recent infection [19, 20].

The three open reading frames (ORFs) altogether forms the HEV genome in which the first, second, and third reading frames encoded proteins ORF1, ORF2, and ORF3 codes for the HEV’s nonstructural polyprotein, capsid, and regulatory protein respectively [21]. Though earlier it was revealed that ORF2 acts as a capsid protein [22], later its role was described in multiple crucial functions of HEV [14, 23–27].

Intrinsically disordered regions (IDRs) [including intrinsically disordered proteins (IDPs) or intrinsically disordered protein regions (IDPRs)] constitute the fraction of a proteome that are known as “dark proteomes.” This dark proteome does not have noticeable similarity to any PDB structure. The IDRs (IDP or IDPR) lack unique structures as they are not folded into three dimensional structures within the viral proteomes [28]. The IDRs exhibit specific functions due to lack of definite 3D structure [29–31]. Additionally, due to possession of intrinsic disorder phenomenon, the IDRs (IDPs or IDPRs) have been correlated with a number of implications in various human diseases (cancer, etc.) [32]. Interestingly, most of the viral proteins possess MoRFs (molecular recognition features), i.e., short protein regions within IDRs that upon binding to partners (interacting partners) undergo disorder-to-order transition [33]. Therefore, the unique characteristics possessed by IDRs assist proteins in their interaction with diverse biological partners and thus form an important requirement for completion of multiple cellular pathway regulation through protein–protein interaction networks [34]. Moreover, the disordered regions in proteins constitute potential drug targets due to their association with important biological processes [35, 36]. As IDP/IDPR is indispensable for carrying out several important crucial functions in viruses, thus they are analyzed using computational approaches [29–31]. It has been revealed recently that besides performing capsid function, the ORF2 plays crucial role in other processes, such as viral replication, immune response regulation, cellular signalling, host tropism, and pathogenesis of HEV. Moreover, it has been suggested that ORF2 has the potential in forming the development of vaccine against HEV [27].

Thus, in this regard, the present study carried out the intrinsic disorder analysis of the HEV ORF2 proteins by employing a set of bioinformatics predictors through evaluating its functional significance. The disorder analysis predicted ORF2 as either moderately disordered or highly disordered protein revealing them as IDPR or IDP variants. The presence of MoRF regions in ORF2 sequences revealed its propensity to bind to interacting partners. Additionally, we also carried out the structure-based analysis of ORF2 protein (using sequences obtained from different host and genotypes) in order to reveal its molecular functions. The identified ion binding, protein binding, and metal binding sites in conjunction with diverse biological processes, such as viral replication, RNA biosynthetic process in the ORF2 protein signified its importance in interaction with the host cell membrane. Our study could shed some novel light on the understanding more ORF2 protein functions beyond its role as a viral capsid protein.

## Methods

### Sequence retrieval

The HEV ORF2 protein sequences were obtained from GenBank at NCBI (National Center for Biotechnology Information). The present analysis included those sequences that encompassed different GTs and hosts and are listed in Table 1.

**Table 1:** Secondary structure and disorder prediction by Phyre2

Sequence	Secondary structure and disorder prediction		
JF443720	Disordered (49%)	Alpha helix (13%)	Beta strand (36%)
M74506	Disordered (50%)	Alpha helix (11%)	Beta strand (36%)
AB222182	Disordered (46%)	Alpha helix (10%)	Beta strand (39%)
GU119961	Disordered (42%)	Alpha helix (15%)	Beta strand (33%)
AB573435	Disordered (44%)	Alpha helix (13%)	Beta strand (38%)
AB602441	Disordered (45%)	Alpha helix (13%)	Beta strand (38%)
KJ496143	Disordered (42%)	Alpha helix (13%)	Beta strand (38%)
KX387865	Disordered (51%)	Alpha helix (11%)	Beta strand (37%)

### Three dimensional (3D) structure

The elements of secondary structure and 3D tertiary structure of the HEV ORF2 proteins were predicted using Phyre2 (*P*rotein *H*omology/Analog*Y R*ecognition *E*ngine) (PHYRE2 Protein Fold Recognition Server (ic.ac.uk) and I-TASSER (Iterative Threading ASSEmbly Refinement) (itasser - Search (bing.com)) webserver, respectively.

### Disorder plot evaluation

The disorder character of the HEV ORF2 proteins was examined using the online tool PONDR (Predictor of Natural Disordered Regions) by considering its default parameters. Different predictors of PONDR like PONDR-VLS2 [37], PONDR-VL3 [38], and PONDR-VL-XT [39], in combination with DISOPRED3 (PSIPRED Workbench (ucl.ac.uk)), were used to identify the disordered regions in the ORF2 proteins.

### MoRFs (molecular recognition features) prediction

The disorder-based binding residues within ORF2 protein segments were identified using three different online predictors, DISOPRED3 (PSIPRED Workbench (ucl.ac.uk)), IUPred3 (IUPred3 (elte.hu)), and IUPred2A (IUPred2A (elte.hu)). The cutoff score was set as 0.5 for all the predictors (cutoff = ≥ 0.5).

### Phosphorylation pattern evaluation

DEPP (disorder enhanced phosphorylation prediction) (http://www.pondr.com/cgi-bin/depp.cgi), an online predictor, was used to predict the phosphorylated residues (Ser, Thr and Tyr) in the HEV ORF2 sequences.

### Structure-based function prediction

COFACTOR algorithm [40, 41] was employed to identify the probable molecular functions and biological processes of the HEV ORF2 proteins by utilizing their 3D structured models.

## Results

**Fig. 1:**
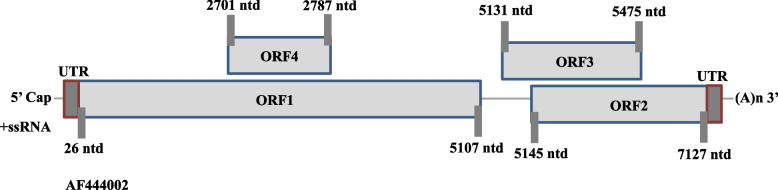
Schematic representation of the genome of HEV. The genomic organization is organized into three open reading frames (ORFs), i.e., ORF1, ORF2, and ORF3. The nucleotide numbers are with reference to the strain Sar55 (GenBank Accession Number: AF444002)

The HEV genome encoded ORF2 starts at the 5147^th^ nucleotide and terminates at the 7127^th^ position of nucleotide. The schematic illustration of the genome of HEV (with reference to Sar55 strain with accession number AF444002) is represented in Fig. 1 [42].

### 3D structures with predicted disorder

**Fig. 2:**
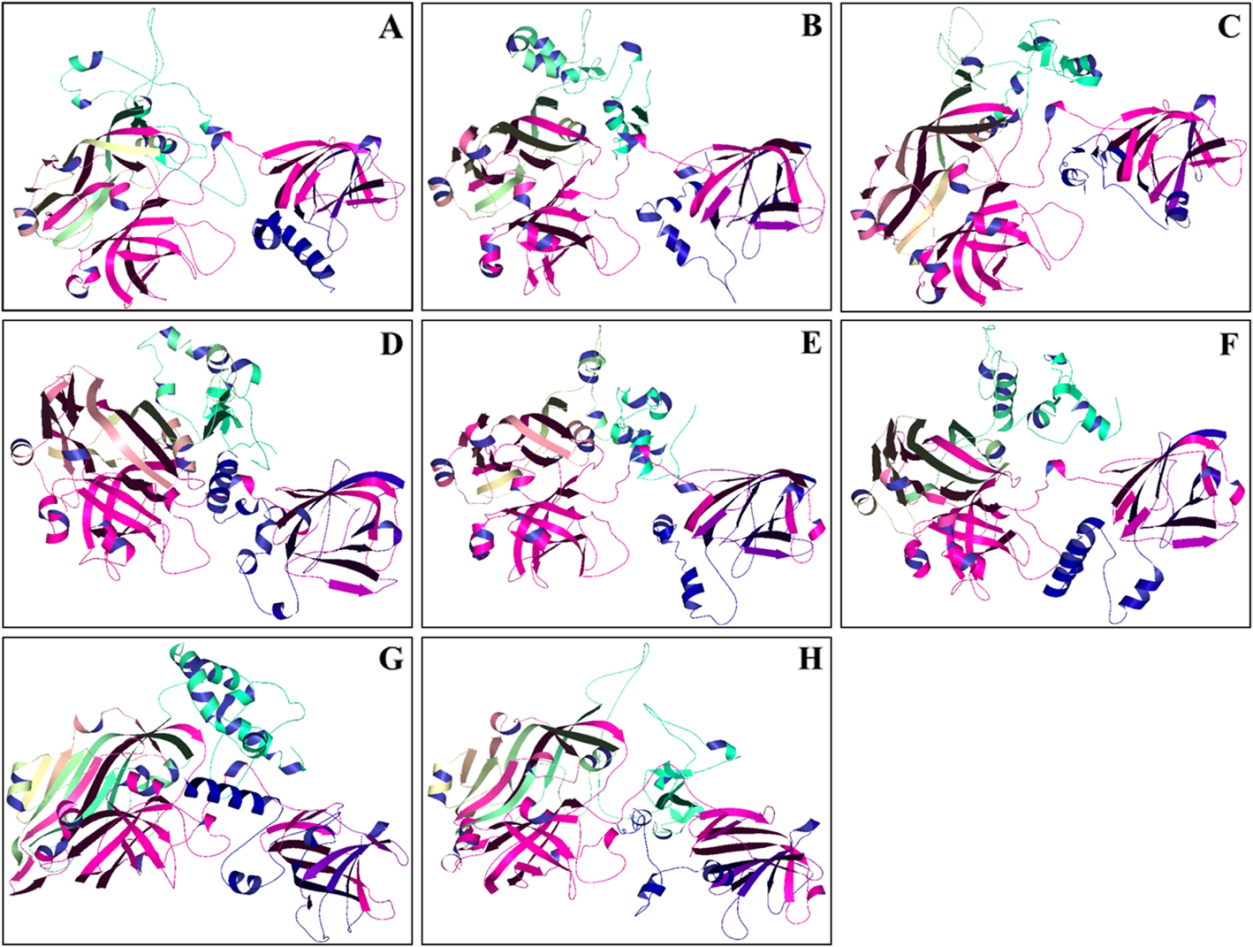
Generated 3D models of the HEV ORF2. **A** JF443720 (GT 1); **B** M74506 (GT 2); **C** AB222182 (GT 3); **D** GU119961 (GT 4); **E** AB573435 (GT 5); **F** AB602441 (GT 6); KJ496143 (GT 7); and **H** KX387865 (GT 8). The prediction was carried out using I-TASSER

The HEV ORF2 protein 3D structures modeled through I-TASSER (based on homology modeling) consisted of alpha helix, beta strand and coil (Fig. 2A–H). The results showed the dominance of coils in comparison to both helices and strands. The predicted percentage of helices, strands, and disorder (evaluated through Phyre2) are mentioned in Table 1.

Thus, our results clearly revealed that the HEV ORF2 proteins consisted of significant fraction of disordered regions. This further prompted us to analyze its intrinsic disorder content using computational approach.

### Intrinsic disorder distribution

The HEV ORF2 proteins intrinsic disorder analysis was evaluated (using different disorder predictors) as mentioned in Table 2. The disorder graphs of the ORF2 proteins are shown in Fig. 2A–H. As suggested, the proteins are termed as structured, moderately disordered, or highly disordered due to their overall predicted intrinsic disorder fraction [43]. Additionally, disorder variants like ORDP (ordered protein), IDPR (intrinsically disordered protein region), and IDP (intrinsically disordered protein) are termed as due to the disordered domain’s length and disordered residue (overall) fraction [44, 45].

**Table 2:** The predicted percentage of intrinsic disorder scores of the ORF2 protein in hepatitis E viruses

Disordered regions	Overall percent disordered	Number of disordered residues	Longest disordered region
**JF443720 [659 AA]**			
**VLXT**			
**[1-4]** MRPR **[21-40]** PPGQPSGRRRGRRSGGSGGG **[65-123]** VTAAAGAGPRVRQPVRPLGSAWRDQAQRPAAASRRRPTTAGAAPLT AVAPAHDTPPVPD **[131-156]** LRRQYNLSTSPLTSSVATGTNLVLYA **[167-181]** DGTNTHIMATEASNY **[186-198]** VARATIRYRPLVP **[219-245]** TSVDMNSVTSTGVRILVQPGIASELVI **[257-289]** WRPVETSGVAEEEATSGLVMLCIHGSPVNSYTN **[316-331]** NTRVSRYSSTARHRLR **[407-412]** PTVKLY **[424-428]** IAIPH **[444-454]** NQHEQDRPTPS **[456-456]** A **[484-502]** YGSSTGPVYVSDSVTLVDD **[577-602]** RVAISTYTTSLGAGPVSISAVAVLDD **[655-655]** K **[657-659]** REL	43.25	17	59
**VL3**			
**[1-56]** MRPRPILLLFLMFLPMLPAPPPGQPSGRRRGRRSGGSGGGFWGDRVDSQPFAPYIH **[65-132]** VTAAAGAGPRVRQPVRPLGSAWRDQAQRPAAASRRRPTTAGAAPLTAVAPAHDTPPVPDVDSRGAILR **[316-338]** NTRVSRYSSTARHRLRRGADGTA **[433-462]** GESRVVIQDYDNQHEQDRPTPSPAPSRPFS **[649-659]** LKMKVGKTREL	28.53	5	68
**VSL2**			
**[1-132]** MRPRPILLLFLMFLPMLPAPPPGQPSGRRRGRRSGGSGGGFWGDRVDSQPFAPYIHPTNPFAPDVTAAAGAGPRVRQPVRPLGSAWRDQAQRPAAASRRRPTTAGAAPLTAVAPAHDTPPVPDVDSRGAILR **[134-134]** Q **[136-145]** NLSTSPLTSS **[216-225]** PTPTSVDMNS **[263-269]** SGVAEEE **[311-341]** TPGNTNTRVSRYSSTARHRLRRGADGTAELT **[440-462]** QDYDNQHEQDRPTPSPAPSRPFS **[480-491]** DQSTYGSSTGPV **[651-659]** MKVGKTREL	35.66	9	132
**DISOPRED3**			
**[1-44]** MRPRPILLLFLMFLPMLPAPPPGQPSGRRRGRRSGGSGGGFWGD **[69-71]** AGA **[73-115]** PRVRQPVRPLGSAWRDQAQRPAAASRRRPTTAGAAPLTAVAPA **[120-121]** PV **[218-219]** PT **[448-458]** QDRPTPSPAPS **[603-618]** HSALALLEDTLDYPAR **[647-659]** QRLKMKVGKTREL	20.33	134	45
**M74506 [659 AA]**			
**VLXT**			
**[1-5]** MRPRP **[21-40]** PTGQPSGRRRGRRSGGTGGG **[64-123]** DVAAASGSGPRLRQPARPLGSTWRDQAQRPSAASRRRPATAGAAALTAVAPAHDTSPVP **[132-182]** LRRQYNLSTSPLTSSVASGTNLVLYAAPLNPPLPLQDGTNTHIMATEASNY **[187-199]** VARATIRYRPLVP **[223-246]** DMNSITSTDVRILVQPGIASELVI **[259-290]** RSVETSGVAEEEATSGLVMLCIHGSPVNSYTN **[321-336]** SRYSSTARHSARGADG **[407-412]** PTVKLY **[446-454]** HEQDRPTPS **[456-456]** A **[486-502]** SSTGPVYISDSVTLVNV **[581-602]** STYTTRLGAGPVAISAAAVLAP **[649-659]** LKVKVGKTREL	43.55	14	60
**VL3**			
**[1-50]** MRPRPLLLLFLLFLPMLPAPPTGQPSGRRRGRRSGGTGGGFWGDRVDSQP **[67-146]** AAASGSGPRLRQPARPLGSTWRDQAQRPSAASRRRPATAGAAALTAVAPAHDTS PVPDVDSRGA ILRRQYNLST SPLTSS **[431-460]** DLGDSRVVIQDYDNQHEQDRPTPSPAPSRP **[654-659]** GKTREL	25.19	4	80
**VSL2**			
**[1-150]** MRPRPLLLLFLLFLPMLPAPPTGQPSGRRRGRRSGGTGGGFWGDRVDSQPFAIPYIHPTNPFAPDVAAASGSGPRLRQPARPLGSTWRDQAQRPSAASRRRPATAGAAALTAVAPAHDTSPVPDVDSRGAILRRQYNLSTSPLTSSVASG **[165-167]** PLQ **[219-226]** PTSVDMNS **[263-270]** TSGVAEEE **[316-342]** TNTRVSRYSSTARHSARGADGTAELTT **[432-432]** L **[435-436]** SR **[439-462]** IQDYDNQHEQDRPTPSPAPSRPFS **[481-490]** QSTYGSSTGP **[652-659]** KVGKTREL	36.57	10	150
**DISOPRED3**			
**[1-39]** MRPRPLLLLFLLFLPMLPAPPTGQPSGRRRGRRSGGTGG **[69-116]** ASGSGPRLRQPARPLGSTWRDQAQRPSAASRRRPATAGAAALTAVAPA **[118-123]** DTSPVP **[132-135]** LRRQ **[217]** T **[219-220]** PT **[222]** V **[359]** G **[453]** P **[455-456]** PA **[603-618]** RSALALLEDTFDYPGR **[647-659]** QRLKVKVGKTREL	20.48	135	48
**AB222182 [660 AA]**			
**VLXT**			
**[1-2]** MR **[19-40]** AGQPSGRRRGRRSGGAGGG **[69-125]** QSGAGARPRQPPRPLGSAWRDQ SQRPSAPPRRQRSTPAGAAPLTAISPAPDTAPVPDV **[132-179]** LRRQYNLSTSPLTSSVASGTNLVLYAAPLNPLLPLQDGTNTHIMATEA **[188-199]** VRATIRYRPLVP **[223-246]** DMNSITSTDVRILVQPGIASELVI **[259-290]** RSVETTGVAEEEATSGLVMLCIHGSPVNSYTN **[318-332]** TRVSRYTSTARHRLR **[408-413]** PTVKLY **[447-455]** HEQDRPTPS **[457-457]** A **[579-609]** VAISTYTTSLGAGPTSISAVGVLAPHSALAV **[654-660]** VGKTRES	40.30	13	57
**VL3**			
**[1-46]** MRPGAVLLLLLVFLPMLPAPPAGQPSGRRRGRRSGGAGGGFWGDRV **[65-140]** DVVSQSGAGARPRQPPRPLGSAWRDQSQRPSAPPRRRSTPAGAAPLTAISPAPDTAPVPDVDSRGAILRRQYNLST **[318-343]** TRVSRYTSTARHRLRRGADGTAELTT **[432-461]** DLGDSRVVIQDYDNQHEQDRPTPSPAPSRP **[651-660]** KMKVGKTRES	28.48	5	76
**VSL2**			
**[1-56]** MRPGAVLLLLLVFLPMLPAPPAGQPSGRRRGRRSGGAGGGFWGDRVDSQPFALPYI **[58-148]** PTNPFVADVVSQSGAGARPRQPPRPLGSAWRDQSQRPSAPPRRRSTPAGAAPLTAISPAPDTAPVPDVDSRGAILRRQYNLSTSPLTSSVA **[218-226]** TPTSVDMNS **[265-270]** GVAEEE **[312-344]** PGNTNTRVSRYTSTARHRLRRGADGTAELTTT **[433-463]** LGDSRVVIQDYDNQHEQDRPTPSPAPSRPFS **[484-484]** T **[486-491]** YGSSTNP **[589-591]** GAG **[652-660]** MKVGKTRES	37.12	10	91
**DISOPRED3**			
**[1-41]** MRPGAVLLLLLVFLPMLPAPPAGQPSGRRRGRRSGGAGGGF **[70-117]** SGAGARPRQPPRPLGSAWRDQSQRPSAPPRRRSTPAGAAPLTAISPAP **[123]** V **[449-458]** QDRPTPSPAP **[605-618]** SALAVLEDTADYPA **[657-660]** LQRLKMKVGKTRES	19.39	128	48
**GU119961 [674 AA]**			
**VLXT**			
**[32-54]** PAPPAGQPSGRRRGRRSSGTGGG **[80-139]** ISTAAGAGARPRQPARPLGSAWRDQSQRPAASSRRRSAPAGASPLTAVAPAPDTAPVPDI **[147-196]** RRQYNLSTSPLTST IATGTNLVLYAAPLSPLLPLQDGTNTHIMATEASNY **[201-213]** VARATIRYRPLVP **[237-260]** DMNSITSTDVRILVQPGIASELVI **[273-304]** RSVETSGVAEEEATSGLVMLCIHGSPVNSYTN **[331-352]** NTRVSRYSSSARHKLRRGPDGT **[422-427]** PTVKLY **[439-443]** IAIPH **[459-469]** NQHEQDRPTPS **[471-471]** A **[598-619]** YTTNLGSGPVSISAVGVLAPHS **[674-674]** Y	40.06	13	60
**VL3**			
**[22-62]** FLLLVLLPMLPAPPAGQPSGRRRGRRSSGTGGGFWGDRVDS **[81-144]** STAAGAGARPRQPARPLGSAWRDQSQRPAASSRRRSAPAGASPLTAVAPA PDTAPVPDIDSRGA **[330-358]** TNTRVSRYSSSARHKLRRGPDGTAELTTT **[448-475]** GESRVVIQDYDNQHEQDRPTPSPAPSRP	24.04	4	64
**VSL2**			
**[1-6]** MNNMFS **[23-25]** LLL **[28-158]** LPMLPAPPAGQPSGRRRGRRSSGTGGGFWGDRVDSQPFALPYIHPTNPFASDISTAAGAGARPRQPARPLGSAWRDQSQRPAASSRRRSAPAGASPLTAVAPAPDTAPVPDIDSRGAILRRQYNLSTSPLT **[233-240]** PTSVDMNS **[277-284]** TSGVAEEE **[326-357]** TPGNTNTRVSRYSSSARHKLRRGPDGTAELTTT **[455-476]** QDYDNQHEQDRPTPSPAPSRPF **[500-505]** GSSTNP **[667-674]** KVGKTREY	33.23	9	131
**DISOPRED3**			
**[1-53]** MNNMFSCSVHGDATMRSRALLFLLLVLLPMLPAPPAGQPSGRRRGRRSSGTGG **[83-130]** AAGAGARPRQPARPLGSAWRDQSQRPAASSRRRSAPAGASPLTAVAPA **[133-135]** TAP **[463-472]** QDRPTPSPAP **[619-633]** SVLAALEDTVDYPAR **[661-674]** LQRLKMKVGKTREY	21.22	143	53
**AB573435 [674 AA]**			
**VLXT**			
**[26-54]** VLLPMLPAPPAGQSSGRRRGRRSGGAGSG **[85-140]** GTGARSRQSARPLGSAWRDQTQRPPAASRRRSTPTGASPLTAVAPAPDTRPVPDVD **[142-142]** R **[145-193]** ILRRQYNLSTSPLTSTIASGTNLVLYAAPLSPLLPLQDGTNTHIMATEA **[202-213]** VRATIRYRPLVP **[236-260]** VDMNSITSTDVRIVVQPGLASELVI **[273-304]** RSVETSGVAEEEATSGLVMLCIHGSPVNSYTN **[331-344]** NTRVSRYSSTARHR **[422-427]** PTVKLY **[461-469]** HEQDRPTPS **[597-618]** TYTTSLGSGPVSVSGVGVLAPH **[665-670]** KMRVGK **[672-672]** R **[674-674]** F	39.02	14	56
**VL3**			
**[27-60]** LLPM LPAPPAGQSSGRRRGRRSGGAGSGFWGDRV **[84-151]** TGTGARSRQSARPLGSAWRDQTQRPPAASRRRSTPTGASPLTAVAPA PDTRPVPDVDSRGAILRRQYN **[333-354]** RVSRYSSTARHRLHRGADGTAE **[449-472]** DSRVVIQDYDNQHEQDRPTPSPAP **[673-674]** EF	22.26	5	68
**VSL2**			
**[1-2]** MN **[32-161]** PAPPAGQSSGRRRGRRSGGAGSGFWGDRVDSQPFALPYIHPTNPFASDTIAATGTGARSRQSARPLGSAWRDQTQRPPAASRRRSTPTGASPLTAVAPAPDTRPVPDVDSRGAILRRQYNLSTSPLTSTIASGTNLVLYAAPLSPLLPLQDGTNTHIMATEA **[232-240]** TPTSVDMNS **[277-284]** TSGVAEEE **[327-357]** PGNTNTRVSRYSSTARHRLHRGAD **[455-476]** QDYDNQHEQDRPTPSPAPSRPF **[501-504]** TSTD **[602-608]** LGSGPVS **[668-674]** VGKTREF	32.64	9	130
**DISOPRED3**			
**[1-55]** MNNMFLCFACGYATMRPRAILLLLVVLLPMLPAPPAGQSSGRRRGRRSGGAGSGF **[84-86]** TGT **[89-237]** RSRQSARPLGSAWRDQTQRPPAASRRRSTPTGASPLTAVAPAPDTRPVP **[462-472]** EQDRPTPSPAP **[618-633]** HAALAVLEDTVDYPAR **[662-674]** QRLKMRVGKTREF	21.81	147	55
**AB602441 [660 AA]**			
**VLXT**			
**[1-2]** MR **[18-40]** PAPPAGQPSGRRRGRRSGGSGGG **[66-67]** VS **[69-125]** SAGAGARARQAARPLGSAWRDQSQRPSASARRRPTPAGASPLTAVAPAPDTTPVPDV **[132-179]** LRRQYNLSTSPLTSTVASGTNLVLYAAPLGPLLPLQDGTNTHIMATEA **[188-199]** IRATIRYRPLVP **[223-246]** DMNSITSTDVRILVQPGLASELII **[259-290]** RSVETSGVAEEEATSGLVMLCIHGSPVNSYTN **[318-332]** TRVSRYTSTARHRLR **[408-413]** PTVKLY **[424-430]** GIAIPHE **[446-455]** QHEQDRPTPS **[582-612]** STYTTNLGSGPVSISAVGVLAPHAATAALED **[652-656]** MKVGK **[658-660]** REF	41.97	15	57
**VL3**			
**[1-52]** MRPRAVLLLFLMLLPMLPAPPAGQPSGRRRGRRSGGSGGGFWGDRVDSQPFA **[68-138]** TSAGAGARARQAARPLGSAWRDQSQRPSASARRRPTPAGASPLTAVAPAPDTTPVPDVDSRGAILRRQYNL **[317-341]** NTRVSRYTSTARHRLRRGPDGTAE L **[432-459]** DLGDSRVTIQDYDNQHEQDRPTPSPAPS **[659-660]** EF	26.97	5	71 0.3317
**VSL2**			
**[1-147]** MRPRAVLLLFLMLLPMLPAPPAGQPSGRRRGRRSGGSGGGFWGDRVDSQPFALPYIHPTNPFASDVSTSAGAGARARQAARPLGSAWRDQSQRPSASARR RPTPAGASPLTAVAPAPDTTPVPDVDSRGAILRRQYNLSTSPLTSTV **[218-226]** TPTSVDMNS **[263-270]** TSGVAEEE **[312-343]** TPGNTNTRVSRYTSTARHRLRRGPDGTAELTT **[432-462]** DLGDSRVTIQDYDNQHEQDRPTPSPAPSRPF **[486-490]** GSTTN **[609-619]** ALEDTADSPAR **[652-660]** MKVGKSREF	38.18	8	147
**DISOPRED3**			
**[1-38]** MRPRAVLLLFLMLLPMLPAPPAGQPSGRRRGRRSGGSG **[70-72]** AGA **[75-117]** RARQAARPLGSAWRDQSQRPSASARRRPTPAGASPLTAVAPAP **[121-122]** PV **[449-458]** QDRPTPSPAP **[604-619]** HAATAALEDTADSPAR **[647-660]** LQRLKMKVGKSREF	18.93	125	43
**KJ496143 [660 AA]**			
**VLXT**			
**[1-2]** MR **[13-40]** LLPMLPAPPAGQSSGRRRGRRSGGSGGG **[71-178]** RSGTGLRQSARPLGTAWRDQSQRPPASTRRRSAPSGAAPLTAVAPAPGTAPVPDVDSRGAVLRRQYNLSTSPLTSTVASGTNLVLYAAPLNPLLPLQDGT NTHIMATE **[188-199]** VRATIRYRPLVP **[222-246]** VDMNSITSTDVRIVVQPGLASELVI **[259-290]** RSVETSGVAEEEATSGLVMLCVHGSPVNSYTN **[317-335]** NTRVSRYSSTARHRLKRGA **[396-398]** FYS **[407-413]** EPSVKLY **[425-429]** IAIPH **[445-455]** NQHEQDRPTPS **[457-457]** A **[580-599]** AISTYTTSLGAGPVAISCVG **[660-660]** Y	41.52	14	108
**VL3**			
**[1-49]** MRPRAILLLLLLLLPMLPAPPAGQSSGRRRGRRSGGSGGGFWGDRVDSQ **[70-142]** SRSGTGLRQSARPLGTAWRDQSQRPPASTRRRSAPSGAAPLTAVAPAPGTAPVPDVDSRGAVLRRQYNLSTSP **[319-340]** RVSRYSSTARHRLKRGADGTAV **[433-461]** LGESRVVIQDYDNQHEQDSRPTPSPAPSRP	26.21	4	73
**VSL2**			
**[1-56]** MRPRAILLLLLLLLPMLPAPPAGQSSGRRRGRRSGGSGGGFWGDRVDSQPFALPYI **[58-147]** PTNPFAADVSAASRSGTGLRQSARPLGTAWRDQSQRPPASTRRRSAPSGAAPLTAVAPAPGTAPVPDVDSRGAVLRRQYNLSTSPLTSTV **[218-226]** TPTSVDMNS **[263-271]** TSGVAEEEA **[313-339]** PGNTNTRVSRYSSTARHRLKRGADGTA **[404-409]** ANGEPS **[433-433]** L **[436-437]** SR **[441-462]** QDYDNQHEQDRPTPSPAPSRPF **[486-491]** GSSTDP **[613-619]** TTDHPAR **[653-660]** KVGKTREY	36.82	12	90
**DISOPRED3**			
**[1-39]** MRPRAILLLLLLLLPMLPAPPAGQSSGRRRGRRSGGSGG **[70-116]** SRSGTGLRQSARPLGTAWRDQSQRPPASTRRRSAPSGAAPLTAVAPA **[448-458]** EQDRPTPSPAP **[606-619]** ALAVLEDTTDHPAR **[647-660]** QRLKMKVGKTREY	18.78	124	46
**KX387865 [660 AA]**			
**VLXT**			
**[19-40]** APPAGQPSGRRRGRRSGGSGGG **[66-129]** ITSSSGAGSRSRQPSRPLGTAWRDQ SQRPAAPTRRRSTPAGAAPLTATAPASGTTPVPDVDSRG **[131-179]** ILRRQYNLSTSPLTSSIASGTNLVLYAAPLSPLLPLQDGTNTHIMATEA **[188-199]** VRATIRYRPLVP **[223-246]** DMNSITSTDVRILVQPGIASELVI **[259-290]** RSVETSGVAEEEATSGLVMLCIHGSPVNSYTN **[318-333]** TRVSRYSSTAHHRLKR **[408-413]** PTVKLY **[425-429]** IAIPH **[445-455]** NQHEQDRPTPS **[457-457]** A **[580-601]** AISTYTTSLGVGPVPISCVGVL **[654-660]** DDDDDDD	41.06	13	64
**VL3**			
**[1-52]** MCTRAVLLLFLLLLPMLPAPPAGQSGRRRGRRSGGSGGGFWGDRVDSQPFA **[64-139]** ADITSSSGAGSRSRQPSRPLGTAWRDQSQRPAAPTRRRSTPAGAAPLTATAPASGTTPVPDVDSRGAILRRQYNLS **[323-342]** YSSTAHHRLKRGADGTAELT **[434-460]** GESRVVIQDYDNQHEQDRPTPSPAPSR **[657-660]** DDDD	27.12	5	76
**VSL2**			
**[1-148]** MCTRAVLLLFLLLLPMLPAPPAGQSGRRRGRRSGGSGGGFWGDRVDSQPFALPYIHPTNPFVADITSSSGAGSRSRQPSRPLGTAWRDQSQRPAAPTRRRSTPAGAAPLTATAPASGTTPVPDVDSRGAILRRQYNLSTSPLTSSIA **[218-226]** TPTSVDMNS **[263-270]** TSGVAEEE **[314-343]** GNTNTRVSRYSSTAHHRLKRGADGTAELTT **[441-462]** QDYDNQHEQDRPTPSPAPSRPF **[487-490]** STTN **[653-660]** DDDDDDDD	34.70	7	148
**DISOPRED3**			
**[1-40]** MCTRAVLLLFLLLLPMLPAPPAGQPSGRRRGRRSGGSGGG **[70-117]** SGAGSRSRQPSRPLGTAWRDQSQRPAAPTRRRSTPAGAAPLTATAPAS **[121-122]** PV **[449-454]** QDRPTP **[455-457]** PAP **[605-617]** SALAVLEDTIDYPA **[647-660]** IQRLKMKVGKTRES	19.39	128	47

#### ORF2 protein (JF443720)

The disorder distribution analysis of the ORF2 polypeptide sequence (JF443720) categorized it into a highly disordered protein as it contained >30% of disordered residues (43.25% by VLXT and 35.66% by VSL2), and moderately disordered protein as it contained <30% of disordered residues (28.53% by VL3). Additionally, the inclusion of long disordered domain at N-terminus in the polypeptide sequence, i.e., up to 59 to 132 consecutive amino acid residues, categorized it into IDP, i.e., protein possessing significant fraction of disordered regions (as predicted by VLXT and VSL2) or IDPR, i.e., structured protein possessing intrinsically disordered segments (as predicted by VL3). The DISPOPRED3 predicted the ORF2 as a moderately disordered protein or IDPR as it contained 20.33% (<30%) of disordered residues with long continuous stretch of disordered domain (about 45 amino acid residues).

#### ORF2 protein (M74506)

The disorder distribution analysis of the ORF2 polypeptide sequence (M74506) categorized it into a highly disordered protein as it contained >30% of disordered residues (43.55% by VLXT and 36.57% by VSL2), and moderately disordered protein as it contained <30% of disordered residues (25.19% by VL3). Additionally, the inclusion of long disordered domain at N-terminus in the polypeptide sequence, i.e., up to 60 to 150 consecutive amino acid residues, categorized it into IDP (as predicted by VLXT and VSL2) or IDPR (as predicted by VL3). The DISPOPRED3 predicted the ORF2 as a moderately disordered protein or IDPR as it contained 20.48% (<30%) of disordered residues with long continuous stretch of disordered domain (about 48 amino acid residues).

#### ORF2 protein (AB222182)

The disorder distribution analysis of the ORF2 polypeptide sequence (AB222182) categorized it into a highly disordered protein as it contained >30% of disordered residues (40.30% by VLXT and 37.12% by VSL2), and moderately disordered protein as it contained <30% of disordered residues (28.48% by VL3). Additionally, the inclusion of long disordered domain at N-terminus in the polypeptide sequence, i.e., up to 57 to 91 consecutive amino acid residues, categorized it into IDP (as predicted by VLXT and VSL2) or IDPR (as predicted by VL3). The DISPOPRED3 predicted the ORF2 as a moderately disordered protein or IDPR as it contained 19.39% (<30%) of disordered residues with long continuous stretch of disordered domain (about 48 amino acid residues).

#### ORF2 protein (GU119961)

The disorder distribution analysis of the ORF2 polypeptide sequence (GU119961) categorized it into a highly disordered protein as it contained >30% of disordered residues (40.06% by VLXT and 33.23% by VSL2), and moderately disordered protein as it contained <30% of disordered residues (24.04% by VL3). Additionally, the inclusion of long disordered domain at N-terminus in the polypeptide sequence, i.e., up to 60 to 131 consecutive amino acid residues was also observed, thus was categorized it into IDP (as predicted by VLXT and VSL2) or IDPR (as predicted by VL3). The DISPOPRED3 predicted the ORF2 as a moderately disordered protein or IDPR as it contained 21.22% (<30%) of disordered residues with long continuous stretch of disordered domain (about 53 amino acid residues).

#### ORF2 protein (AB573435)

The disorder distribution analysis of the ORF2 polypeptide sequence (AB5734351) categorized it into a highly disordered protein as it contained >30% of disordered residues (39.02% by VLXT and 32.64% by VSL2). Additionally, the inclusion of long disordered domain at N-terminus in the polypeptide sequence, i.e., up to 56 to 130 consecutive amino acid residues, categorized it into IDP (as predicted by VLXT and VSL2). The DISPOPRED3 predicted the ORF2 as a moderately disordered protein or IDPR as it contained 21.81% (<30%) of disordered residues with long continuous stretch of disordered domain (about 55 amino acid residues).

#### ORF2 protein (AB602441)

The disorder distribution analysis of the ORF2 polypeptide sequence (AB602441) categorized it into a highly disordered protein as it contained >30% of disordered residues (41.97% by VLXT and 38.18% by VSL2), and moderately disordered protein as it contained <30% of disordered residues (26.97% by VL3). Additionally, the inclusion of long disordered domain at N-terminus in the polypeptide sequence, i.e., up to 57 to 147 consecutive amino acid residues, categorized it into IDP (as predicted by VLXT and VSL2) or IDPR (as predicted by VL3). The DISPOPRED3 predicted the ORF2 as a moderately disordered protein or IDPR as it contained 18.93% (<30%) of disordered residues with long continuous stretch of disordered domain (about 43 amino acid residues).

#### ORF2 protein (KJ496143)

The disorder distribution analysis of the ORF2 polypeptide sequence (KJ496143) categorized it into a highly disordered protein as it contained >30% of disordered residues (41.52% by VLXT and 36.82% by VSL2), and moderately disordered protein as it contained <30% of disordered residues (26.21% by VL3). Additionally, the inclusion of long disordered domain at N-terminus in the polypeptide sequence, i.e., up to 90 to 108 consecutive amino acid residues, categorized it into IDP (as predicted by VLXT and VSL2) or IDPR (as predicted by VL3). The DISPOPRED3 predicted the ORF2 as a moderately disordered protein or IDPR as it contained 18.78% (<30%) of disordered residues with long continuous stretch of disordered domain (about 46 amino acid residues).

#### ORF2 protein (KX387865)

The disorder distribution analysis of the ORF2 polypeptide sequence (KX387865) categorized it into a highly disordered protein as it contained >30% of disordered residues (41.06% by VLXT and 34.70% by VSL2), and moderately disordered protein as it contained <30% of disordered residues (27.192 by VL3). Additionally, the inclusion of long disordered domain at N-terminus in the polypeptide sequence, i.e., up to 64 to 148 consecutive amino acid residues, categorized it into IDP (as predicted by VLXT and VSL2) or IDPR (as predicted by VL3). The DISPOPRED3 predicted the ORF2 as a moderately disordered protein or IDPR as it contained 19.39% (<30%) of disordered residues with long continuous stretch of disordered domain (about 47 amino acid residues).

On combining the results, obtained from the aforementioned predictors (VLXT, VL3, VSL2, and DISOPRED3), it was inferred that the HEV exhibited significant intrinsic disorder character in the ORF2 proteins.

### Categorizing protein variant on the basis of predicted disorder percentage

Next we combined the results of obtained disorder predictors for the HEV ORF2 protein sequences to categorize them into a specific category of disorder variant, i.e., ORDP, IPD, or IDPR. The mean PPID (predicted percentage of intrinsic disorder) score was obtained by summing up the individual percentage disorder score predicted by different predictors (VLXT, VL3 VSL2, and DISOPRED3) and dividing by 4. The mean PPID scores of the ORF2 proteins are mentioned in Table 3.

**Table 3:** Categorization of the disorder variant of the HEV ORF2 proteins

Sequences	Mean PPID (%)	Categorization
JF443720	31.94 (>30)	IDP/ Highly disordered protein
M74506	31.44 (>30)	IDP/ Highly disordered protein
AB222182	31.32 (>30)	IDP/ Highly disordered protein
GU119961	29.63 (<30)	IDPR/ Moderately disordered protein
AB573435	28.93 (<30)	IDPR/ Moderately disordered protein
AB602441	31.51 (>30)	IDP/ Highly disordered protein
KJ496143	30.83 (>30)	IDP/ Highly disordered protein
KX387865	30.56 (>30)	IDP/ Highly disordered protein

As it could be interpreted from Table 3, most of the HEV ORF2 were predicted as highly disordered proteins or IDPs due to the presence of more than 30% of the disordered residues in its polypeptide chain. However, two proteins (GU119961 and AB573435) were categorized as moderately disordered proteins or IDPRs as they consisted of less than 30% of the disordered residues in its polypeptide chain along with disordered domains (as seen in Table 2). But it is important to mention that these sequences also possessed IDP character as the percentages were only almost equivalent to 30. All the ORF2 protein sequences obtained from different genotypes showed that significant proportion of their fraction consisted disordered character. Thus, it can be assumed from these results that ORF2 belonged to the IDP category.

### Disorder-based binding site in protein

In addition to intrinsic disorder, we have also computationally estimated the presence of disorder-based binding sites, MoRFs, in each HEV ORF2 protein. The predicted MoRFs for individual ORF2 protein, by different computational tools (DISOPRED3, and IUPRED3 ANCHOR and IUPRED2A ANCHOR), are listed in Table 4.

**Table 4:** Identified MoRF regions in HEV ORF2 proteins

PROTEIN	DISOPRED3 (cutoff = ≥ 0.5)	IUPRED3 ANCHOR (cutoff = ≥ 0.5)	IUPRED2A ANCHOR (cutoff = ≥ 0.5)
JF443720	**[1-11]** MRPRPILLLFL **[25-34]** PSGRRRGRRS **[70-71]** GA **[89-100]** QAQRPAAASRRR **[218-219]** PT **[448-455]** QDRPTPSP **[457-458]** PS **[603-618]** HSALALLEDTLDYPAR **[647-659]** QRLKMKVGKTREL	**[1-21]** MRPRPILLLFLMFLPMLPAPP **[30-136]** RGRRSGGSGGGFWGDRVDSQPFAPYIHPTNPFAPDVTAAAGAGPRVRQPVRPLGSAWRDQAQRPAAASRRRPTTAGAAPLTAVAPAHDTPPVPDVDSRGAILRRQYN **[425-439]** AIPHDIDLGESRVVI	**[1-21]** MRPRPILLLFLMFLPMLPAPP **[30-136]** RGRRSGGSGGGFWGDRVDSQPFAPYIHPTNPFAPDVTAAAGAGPRVRQPVRPLGSAWRDQAQRPAAASRRRPTTAGAAPLTAVAPAHDTPPVPDVDSRGAILRRQYN **[425-439]** AIPHDIDLGESRVVI
M74506	**[1-11]** MRPRPLLLLFLLFL **[21-35]** PTGQPSGRRRGRRSG **[45-49]** SAASR **[121-123]** PVP **[134]** R **[603-618]** RSALALLEDTFDYPGR **[647-659]** QRLKVKVGKTREL	**[2-20]** RPRPLLLLFLLFLPMLPAP **[35-138]** GGTGGGFWGDRVDSQPFAIPYIHPTNPFAPDVAAASGSGPRLRQPARPLGSTWRDQAQRPSAASRRRPATAGAAALTAVAPAHDTSPVPDVDSRGAILRRQYNL **[424-441]** VAIPHDIDLGDSRVVIQD	**[2-20]** RPRPLLLLFLLFLPMLPAP **[35-138]** GGTGGGFWGDRVDSQPFAIPYIHPTNPFAPDVAAASGSGPRLRQPARPLGSTWRDQAQRPSAASRRRPATAGAAALTAVAPAHDTSPVPDVDSRGAILRRQYNL **[424-441]** VAIPHDIDLGDSRVVIQD
AB222182	**[1-11]** MRPGAVLLLLL **[22-35]** AGQPSGRRRGRRSG **[95-96]** SA **[449-454]** QDRPTP **[456-458]** PAP **[605-618]** SALAVLEDTADYPA **[647-660]** LQRLKMKVGKTRES	**[4-18]** GAVLLLLLVFLPMLP **[37-138]** AGGGFWGDRVDSQPFALPYIHPTNPFVADVVSQSGAGARPRQPPRPLGSAWRDQSQRPSAPPRRRSTPAGAAPLTAISPAPDTAPVPDVDSRGAILRRQYNL **[425-442]** ITIPHDIDLGDSRVVIQDYDNQHEQD	**[4-18]** GAVLLLLLVFLPMLP **[37-138]** AGGGFWGDRVDSQPFALPYIHPTNPFVADVVSQSGAGARPRQPPRPLGSAWRDQSQRPSAPPRRRSTPAGAAPLTAISPAPDTAPVPDVDSRGAILRRQYNL **[425-442]** ITIPHDIDLGDSRVVIQDYDNQHEQD
GU119961	**[1-5]** MNNMF **[19-24]** ALLFLL **[42]** R **[44-45]** RG **[106-115]** QRPAASSRRR **[463-468]** QDRPTP **[470-472]** PAP **[619-633]** SVLAALEDTVDYPAR **[661-674]** LQRLKMKVGKTREY	[22-29] FLLLVLLP [53-146] GGFWGDRVDSQPFALPYIHPTNPFASDISTAAGAGARPRQPARPLGSAWRDQSQRPAASSRRRSAPAGASPLTAVAPAPDTAPVPDIDSRGAIL **[439-455]** IAIPHDIDLGESRVVIQ	[22-29] FLLLVLLP [53-146] GGFWGDRVDSQPFALPYIHPTNPFASDISTAAGAGARPRQPARPLGSAWRDQSQRPAASSRRRSAPAGASPLTAVAPAPDTAPVPDIDSRGAIL **[439-455]** IAIPHDIDLGESRVVIQ
AB573435	**[1-5]** MNNMF **[21-24]** LLLL **[85-86]** GT **[462-472]** EQDRPTPSPAP **[618-633]** HAALAVLEDTVDYPAR **[662-674]** QRLKMRVGKTREF	**[56-153]** WGDRVDSQPFALPYIHPTNPFASDTIAATGTGARSRQSARPLGSAWRDQTQRPPAASRRRSTPTGASPLTAVAPAPDTRPVPDVDSRGAILRRQYNLS **[439-454]** GIAIPHDIDLGDSRVVI	**[56-153]** WGDRVDSQPFALPYIHPTNPFASDTIAATGTGARSRQSARPLGSAWRDQTQRPPAASRRRSTPTGASPLTAVAPAPDTRPVPDVDSRGAILRRQYNLS **[439-454]** GIAIPHDIDLGDSRVVI
AB602441	**[1-12]** MRPRAVLLLFLM **[21-35]** PAGQPSGRRRGRRSG **[71-72]** GA **[90]** Q **[92-99]** QRPSASAR **[449-454]** QDRPTP **[456-458]** PAP **[604-619]** HAATAALEDTADSPAR **[447-660]** LQRLKMKVGKSREF	**[2-19]** RPRAVLLLFLMLLPMLPA **[37-139]** SGGGFWGDRVDSQPFALPYIHPTNPFASDVSTSAGAGARARQAARPLGSAWRDQSQRPSASARRRPTPAGASPLTAVAPAPDTTPVPDVDSRGAILRRQYNLS **[424-439]** GIAIPHEIDLGDSRVTI	**[2-19]** RPRAVLLLFLMLLPMLPA **[37-139]** SGGGFWGDRVDSQPFALPYIHPTNPFASDVSTSAGAGARARQAARPLGSAWRDQSQRPSASARRRPTPAGASPLTAVAPAPDTTPVPDVDSRGAILRRQYNLS **[424-439]** GIAIPHEIDLGDSRVTI
KJ496143	**[1-11]** MRPRAILLLLL **[21-35]** PAGQSSGRRRGRRSG **[95-96]** PA **[448-458]** EQDRPTPSPAP **[606-619]** ALAVLEDTTDHPAR **[648-660]** QRLKMKVGKTREY	**[7-17]** LLLLLLLLPML **[42-138]** WGDRVDSQPFALPYIHPTNPFAADVSAASRSGTGLRQSARPLGTAWRDQSQRPPASTRRRSAPSGAAPLTAVAPAPGTAPVPDVDSRGAVLRRQYNL **[426-438]** AIPHDSRV	**[7-17]** LLLLLLLLPML **[42-138]** WGDRVDSQPFALPYIHPTNPFAADVSAASRSGTGLRQSARPLGTAWRDQSQRPPASTRRRSAPSGAAPLTAVAPAPGTAPVPDVDSRGAVLRRQYNL **[426-438]** AIPHDSRV
KX387865	**[1-11]** MCTRAVLLLFL **[22-35]** AGQPSGRRRGRRSG **[96]** A **[449-454]** QDRPTP **[456-458]** PAP **[605-618]** SALAVLEDTIDYPA **[647-660]** IQRLKMKVGKTRES	**[6-16]** VLLLFLLLLP **[41-138]** WGDRVDSQPFALPYIHPTNPFVADITSSSGAGSRSRQPSRPLGTAWRDQSQRPAAPTRRRSTPAGAAPLTATAPASGTTPVPDVDSRGAILRRQYNL **[425-441]** IAIPHDIDLGESRVVIQ	

### Analysis of phosphorylation sites

The ORF2 sequences were predicted with several phosphorylation sites (P-sites). The predicted phosphorylated residues, i.e., Ser, Thr, and Tyr in HEV ORF2 sequences with the DEPP score are summarized in Table 5 (Fig. 4).

**Table 5:** Predicted number and percentage of phosphorylated residues in ORF2 of hepatitis E viruses

Sequences	Number of phosphorylated residues		
	Ser	Thr	Tyr
JF443720	10 out 54 (18.51%)	14 out of 62 (22.58%)	3 out of 24 (12.5%)
M74506	15 out 56 (26.78%)	9 out of 62 (14.51%)	4 out of 23 (17.39%)
AB222182	14 out of 55 (25.45%)	12 out of 67 (17.91%)	3 out of 24 (12.50%)
GU119961	17 out of 62 (27.41%)	11 out of 65 (16.92%)	4 out of 25 (16.00%)
AB573435	16 out of 57 (28.07%)	14 out of 70 (20.00%)	3 out of 24 (12.50%)
AB602441	15 out of 55 (27.27%)	14 out of 66 (21.21%)	3 out of 24 (12.50%)
KJ496143	18 out of 55 (32.72%)	11 out of 64 (17.18%)	6 out of 24 (25.00%)
KX387865	20 out 61 (32.78%)	13 out of 69 (18.84%)	3 out of 23 (13.04%)

**Fig. 3:**
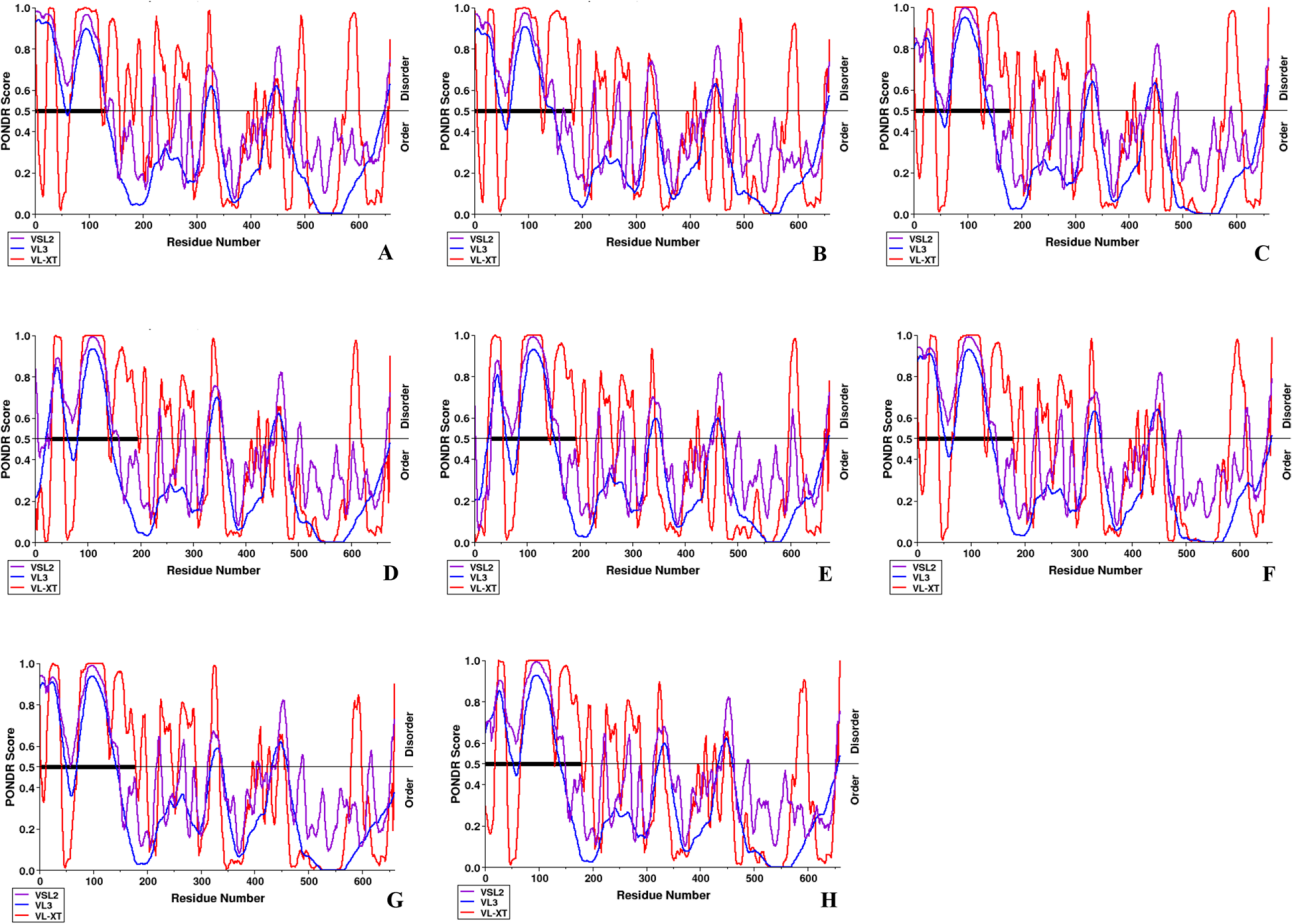
Analysis of intrinsic disorder predisposition of HEV ORF2. **A** JF443720 (GT 1); **B** M74506 (GT 2); **C** AB222182 (GT 3); **D** GU119961 (GT 4); **E** AB573435 (GT 5); **F** AB602441 (GT 6); KJ496143 (GT 7); and **H** KX387865 (GT 8). Graphs **A**–**H** represent the intrinsic disorder profiles of ORF2 sequences of HEV. Disorder probability was calculated using three members of the family PONDR (Prediction of Natural Disordered Regions), i.e., VLXT, VL3, and VSL2. A threshold value of 0.5 was set to distinguish between ordered and disordered region along the genome (line). Regions above the threshold are predicted to be disordered

**Fig. 4:**
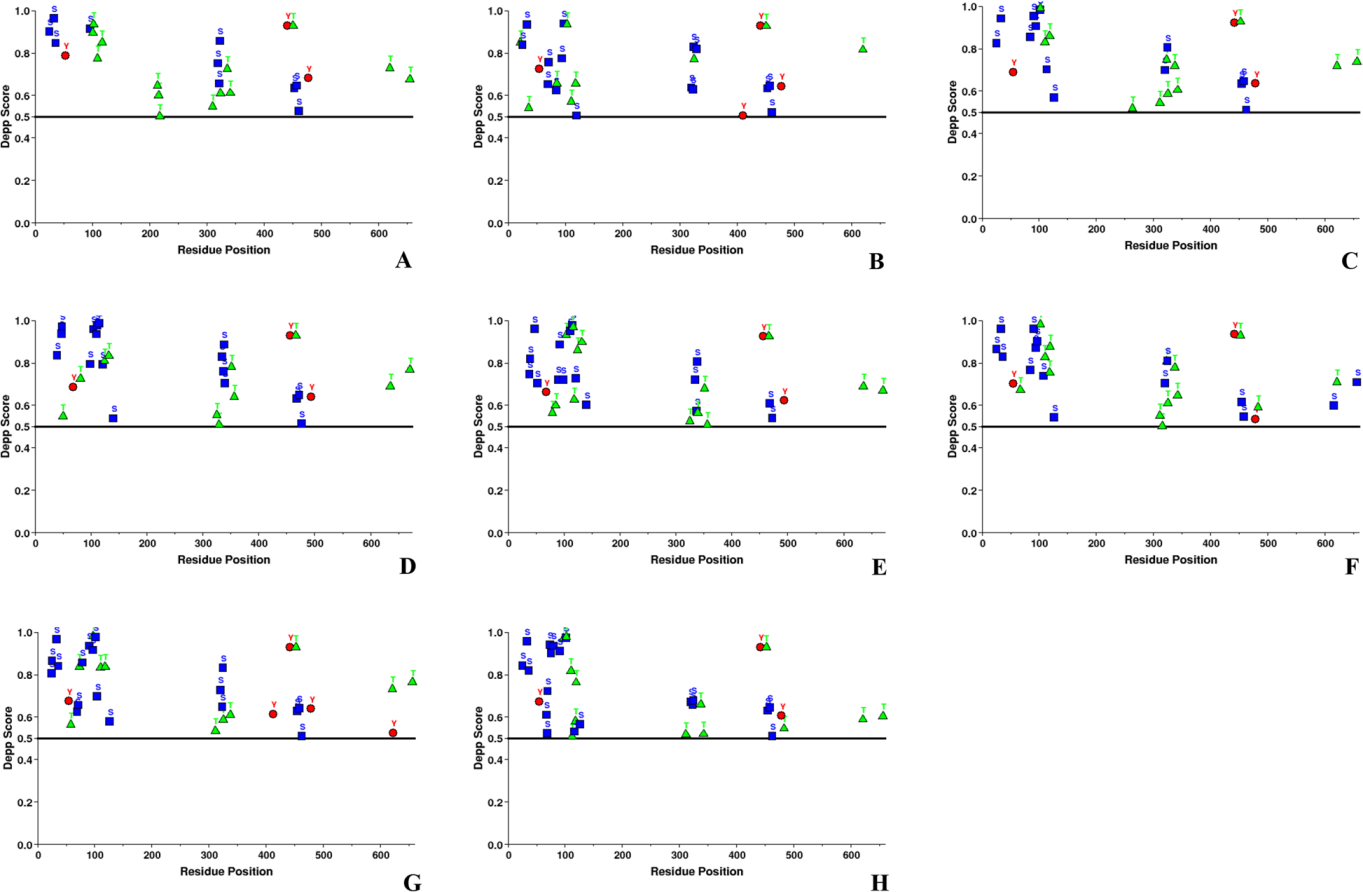
Prediction of phosphorylation sites showing the scores of phosphorylated residues (Ser, Thr, Tyr) along with the depicted scores within ORF2. **A** JF443720 (GT 1); **B** M74506 (GT 2); **C** AB222182 (GT 3); **D** GU119961 (GT 4); **E** AB573435 (GT 5); **F** AB602441 (GT 6); KJ496143 (GT 7); and **H** KX387865 (GT 8). Graphs **A**–**H** represent the phosphorylation patterns of the ORF2 sequences of HEV. The score was computed using DEPP (disorder enhanced phosphorylation prediction). A threshold value of 0.5 was set to distinguish between ordered and disordered region along the genome (line). The predicted phosphorylated residues above the threshold are represented as Ser (S): Blue, Thr (T): Green, and Tyr (Y): Red

Our results revealed that Ser was found in higher fractions in comparison to other phosphorylated residues (Thr and Tyr) (Fig. 4). Our analysis revealed that most of the P-sites were present within the disordered ORF2 regions which clearly indicated the correlation between disordered regions and phosphorylation sites (Figs. 3 and 4).

### Prediction of consensus GO terms

The putative 3D modeled structure-based molecular functions and biological processes (using COFACTOR algorithm) are summarized in Table 6.

**Table 6:** Predicted consensus GO terms for homology modeled ORF2 structures

Consensus GO terms	Description
**JF443720**	
**Molecular function**	
GO:0005198~structural molecule activity	The action of a molecule that contributes to the structural integrity of a complex or its assembly within or outside a cell.
GO:0043167~ion binding	Binding to an ion, a charged atoms or groups of atoms.
**Biological process**	
GO:0005975~carbohydrate metabolic process	The chemical reactions and pathways involving carbohydrates, any of a group of organic compounds based of the general formula Cx(H2O)y.
GO:0006955~immune response	Any immune system process that functions in the calibrated response of an organism to a potential internal or invasive threat.
GO:0006935~chemotaxis	The directed movement of a motile cell or organism, or the directed growth of a cell guided by a specific chemical concentration gradient. Movement may be towards a higher concentration (positive chemotaxis) or towards a lower concentration (negative chemotaxis).
GO:0006260~DNA replication	The cellular metabolic process in which a cell duplicates one or more molecules of DNA.
GO:0034638~phosphatidylcholine catabolic process	The chemical reactions and pathways resulting in the breakdown of phosphatidylcholines, any of a class of glycerophospholipids in which the phosphatidyl group is esterified to the hydroxyl group of choline.
**M74506**	
**Molecular function**	
GO:0005198~structural molecule activity	The action of a molecule that contributes to the structural integrity of a complex or its assembly within or outside a cell.
GO:0043167~ion binding	Binding to an ion, a charged atoms or groups of atoms.
**Biological process**	
GO:0022900~electron transport chain	A process in which a series of electron carriers operate together to transfer electrons from donors to any of several different terminal electron acceptors.
GO:0006810~transport	The directed movement of substances (such as macromolecules, small molecules, ions) or cellular components (such as complexes and organelles) into, out of or within a cell, or between cells, or within a multicellular organism by means of some agent such as a transporter, pore or motor protein.
GO:0019079~viral genome replication	Any process involved directly in viral genome replication, including viral nucleotide metabolism.
GO:0032774~RNA biosynthetic process	The chemical reactions and pathways resulting in the formation of RNA, ribonucleic acid, one of the two main type of nucleic acid, consisting of a long, unbranched macromolecule formed from ribonucleotides joined in 3',5'-phosphodiester linkage. Includes polymerization of ribonucleotide monomers. Refers not only to transcription but also to e.g. viral RNA replication.
GO:0006221~pyrimidine nucleotide biosynthetic process	The chemical reactions and pathways resulting in the formation of a pyrimidine nucleotide, a compound consisting of nucleoside (a pyrimidine base linked to a deoxyribose or ribose sugar) esterified with a phosphate group at either the 3' or 5'-hydroxyl group of the sugar.
**AB222182**	
**Molecular function**	
GO:0005198~structural molecule activity	The action of a molecule that contributes to the structural integrity of a complex or its assembly within or outside a cell.
GO:0016638~oxidoreductase activity, acting on the CH-NH2 group of donors	Catalysis of an oxidation-reduction (redox) reaction in which a CH-NH2 group acts as a hydrogen or electron donor and reduces a hydrogen or electron acceptor.
GO:0046872~metal ion binding	Binding to a metal ion.
GO:0051536 ~ iron-sulfur cluster binding	Binding to an iron-sulfur cluster, a combination of iron and sulfur atoms.
**Biological process**	
GO:0009084~glutamine family amino acid biosynthetic process	The chemical reactions and pathways resulting in the formation of amino acids of the glutamine family, comprising arginine, glutamate, glutamine and proline.
GO:0006536~glutamate metabolic process	The chemical reactions and pathways involving glutamate, the anion of 2-aminopentanedioic acid.
**GU119961**	
**Molecular function**	
GO:0005198~structural molecule activity	The action of a molecule that contributes to the structural integrity of a complex or its assembly within or outside a cell.
GO:0043167~ion binding	Binding to an ion, a charged atoms or groups of atoms.
**Biological process**	
GO:0051234~establishment of localization	Any process that localizes a substance or cellular component. This may occur via movement, tethering or selective degradation.
**AB573435**	
**Molecular function**	
GO:0005198~structural molecule activity	The action of a molecule that contributes to the structural integrity of a complex or its assembly within or outside a cell.
GO:0043167~ion binding	Binding to an ion, a charged atoms or groups of atoms.
**Biological process**	
GO:0051234~establishment of localization	Any process that localizes a substance or cellular component. This may occur via movement, tethering or selective degradation.
**AB602441**	
**Molecular function**	
GO:0005198~structural molecule activity	The action of a molecule that contributes to the structural integrity of a complex or its assembly within or outside a cell.
GO:0043167~ion binding	Binding to an ion, a charged atoms or groups of atoms.
**Biological process**	
GO:0051234~establishment of localization	Any process that localizes a substance or cellular component. This may occur via movement, tethering or selective degradation.
**KJ496143**	
**Molecular function**	
GO:0005198~structural molecule activity	The action of a molecule that contributes to the structural integrity of a complex or its assembly within or outside a cell.
GO:0016726~oxidoreductase activity, acting on CH or CH2 groups, NAD or NADP as acceptor	Catalysis of an oxidation-reduction (redox) reaction in which a CH2 group acts as a hydrogen or electron donor and reduces NAD+ or NADP.
GO:0016727~oxidoreductase activity, acting on CH or CH2 groups, oxygen as acceptor	Catalysis of an oxidation-reduction (redox) reaction in which a CH2 group acts as a hydrogen or electron donor and reduces an oxygen molecule.
GO:0046914~ transition metal ion binding	Binding to a transition metal ions; a transition metal is an element whose atom has an incomplete d-subshell of extranuclear electrons, or which gives rise to a cation or cations with an incomplete d-subshell. Transition metals often have more than one valency state. Biologically relevant transition metals include vanadium, manganese, iron, copper, cobalt, nickel, molybdenum and silver.
GO:0051536~iron-sulfur cluster binding	Binding to an iron-sulfur cluster, a combination of iron and sulfur atoms.
**Biological process**	
GO:0006145~purine nucleobase catabolic process	The chemical reactions and pathways resulting in the breakdown of purine nucleobases, one of the two classes of nitrogen-containing ring compounds found in DNA and RNA, which include adenine and guanine.
GO:0046110~xanthine metabolic process	The chemical reactions and pathways involving xanthine, 2,6-dihydroxypurine, a purine formed in the metabolic breakdown of guanine but not present in nucleic acids.
**KX387865**	
**Molecular function**	
GO:0005198~structural molecule activity	The action of a molecule that contributes to the structural integrity of a complex or its assembly within or outside a cell.
GO:0051540~metal cluster binding	Binding to a cluster of atoms including both metal ions and nonmetal atoms, usually sulfur and oxygen. Examples include iron-sulfur clusters and nickel-iron-sulfur clusters.
GO:0016726~oxidoreductase activity, acting on CH or CH2 groups, NAD or NADP as acceptor	Catalysis of an oxidation-reduction (redox) reaction in which a CH2 group acts as a hydrogen or electron donor and reduces NAD+ or NADP.
GO:0016727~oxidoreductase activity, acting on CH or CH2 groups, oxygen as acceptor	Catalysis of an oxidation-reduction (redox) reaction in which a CH2 group acts as a hydrogen or electron donor and reduces an oxygen molecule.
GO:004280**~**identical protein binding	Binding to an identical protein or proteins.
GO:0046914~transition metal ion binding	Binding to a transition metal ions; a transition metal is an element whose atom has an incomplete d-subshell of extranuclear electrons, or which gives rise to a cation or cations with an incomplete d-subshell. Transition metals often have more than one valency state. Biologically relevant transition metals include vanadium, manganese, iron, copper, cobalt, nickel, molybdenum and silver.
**Biological process**	
GO:0007589~body fluid secretion	The controlled release of a fluid by a cell or tissue in an animal.
GO:0030855~epithelial cell differentiation	The process in which a relatively unspecialized cell acquires specialized features of an epithelial cell, any of the cells making up an epithelium.
GO:0045595~ regulation of cell differentiation	Any process that modulates the frequency, rate or extent of cell differentiation, the process in which relatively unspecialized cells acquire specialized structural and functional features.
GO:0006145~purine nucleobase catabolic process	The chemical reactions and pathways resulting in the breakdown of purine nucleobases, one of the two classes of nitrogen-containing ring compounds found in DNA and RNA, which include adenine and guanine.
GO:2000026~regulation of multicellular organismal development	Any process that modulates the frequency, rate or extent of multicellular organismal development.

The molecular functions included structural molecule activity, ion binding, metal ion binding, transition metal ion binding, ion-sulfur cluster binding, and oxidoreductase activity. In this regard, our gene ontology findings clearly revealed that binding interactions in conjunction with catalytic activities were attributed to ORF2. The binding interactions, such as metal cluster binding (GO:0051540), protein binding (GO:004280), transition metal ion binding (GO:0046914), and iron-sulfur cluster binding (GO:0051536) revealed the propensity of ORF2 to bind to a variety of molecules (ion, metal, protein). Furthermore, the involvement of ORF2 proteins in different predicted biological processes, such as, electron transport chain (GO:0022900), oxidoreductase activity (GO:0016638), DNA replication (GO:0006260), and cell differentiation regulation (GO:0045595) revealed the significant mitochondrial functions as well as significant processes attributed to ORF2 (Table 6).

## Discussion

The three ORFs (ORF1, ORF2 and ORF3) constitute the genome of HEV [21]. The ORF2 encoded protein polypeptide comprises 660 amino acids [46] and codes for the viral capsid [22, 23]. The intrinsic disorder occurrence in diverse viral proteins has been predicted through different bioinformatics tools [47–49]. The disordered segments (IDRs) in viral proteins perform indispensable functions like accommodation and adaptation of the virus in unsympathetic habitats, and assist in helping proper management of virus and invasion of the host cell pathways [50, 51]. IDPs are often frequently associated with the progression of diseases and they constitute druggable-targets [35, 36, 52, 53]. The ORF2 protein performs various regulatory roles in addition to its role in viral replication and pathogenesis [54]. Also, its application in vaccine development has been documented recently [54]. Thus, targeting the ORF2 protein is ideal for devising treatment against the HEV. Some of our recent investigations have shown varied levels of disorderedness in different HEV ORF encoded proteins [55–60], however, irrespective of the significance of the ORF2 protein role in the virus life cycle, its disorder character has not been explored in different GTs and hosts. In this regard, it is essential to investigate the disorder status of the ORF2 protein (sequences that encompassed different hosts and genotypes) to understand its functions based on its disorderness. The presented study employed different computational tools to shed light on the ORF2 disorderedness in HEV functionality through utilizing GenBank data.

As detailed examination of a protein’s structure provides knowledge on its functional aspects, therefore, we scrutinized the homology modeled ORF2 structures generated through I-TASSER webserver (a portal for protein modeling and analysis). The homology modeled structures comprised major secondary elements (in form of α-helix, β-strand) and coils. As defined by Kabsch and Sander in 1983 [61], in a study [62], that though coils/loops are not necessarily found within disorder protein segments, necessarily disorderness of proteins exists in loops only [62]. On examining the ORF2 constructed homology models, we found that the structures possessed significant disordeness that initially revealed ORF2 proteins with significant percentage of IDRs within loops. Further, detailed examination of the ORF2 proteins was undertaken by employing various disorder predictors. The presented study utilized three PONDR family members VLXT, VL3, and VSL2 [37–39], to examine the ORF2 proteins related to HEV. The predictor VL3 was chosen as it predicts disorderness of long segments with high accuracy [63], whereas VLXT was shown as it is very sensitive [64, 65]. DISOPRED3 was utilized as it predicts disordered segments within protein sequences precisely [66].

The complete life cycle of a virus is achieved by establishing a variety of interactions with the different components of host cell. The various stages of virus life cycle, such as, its attachment, entry, commandeering host machinery, viral component synthesis, and assembly till its exit from hosts as new infectious particle, heavily depend on the intrinsic disorder prevalent in their proteomes [67]. Importantly, studies have shown the relation of intrinsically disorder protein to specific roles [68] in viruses like HCV (hepatitis C virus) [69], MeV (Measles virus) [70], and Hendra virus [71]. The nonstructural HEV ORF1 domains like PPR (Polyproline region) [72] and Y-domain [55] in addition to other proteins [56–60] have also been linked to regulation of HEV due to its characteristic intrinsic disorder property. The HEV ORF2 proteins were initially categorized into structured proteins, moderately disordered proteins, and highly disordered proteins, on the basis of the overall degree of intrinsic disorder [43]. Next, the ORF2 proteins were categorized into ORDPs, IDPRs, and IDPs on the basis of the disordered domain’s length with the overall disordered fraction of residues [44, 45]. These three categories of intrinsic disorder variants are briefly described as follows: ORDPs are variants which consist of less than 30% of disordered residues with the absence of disordered domain at either terminus (C- or N-) or in positions distinct from both terminals. IDPRs are variants which consist of less than 30% of disordered residues with the presence of disordered domain at either terminus (C- or N-) or in positions distinct from the terminals. IDPs are variants which consist of more than 30% of disordered residues. On exploiting these criteria, our intrinsic disorder propensity analysis revealed that ORF2 as a highly disordered protein or moderately disordered protein categorizing them as IDP or IDPR. Our results showed the disorderness in ORF2 protein at N-terminals is due to the occurrence of continuous long disordered domains. According to a study, it has been revealed that the N-terminal region arginine-rich motif (from 1st to 111th amino acid residues) of the ORF2 protein inhibits the phosphorylation of IRF3 (Interferon Regulatory Factor 3) via interacting with a multiprotein complex [73]. But the exact domain binding this complex remains to be determined. A recent study has shown the involvement of arginine-rich motifs in nuclear translocation of ORF2 by serving as nuclear location signals [74]. Thus, it is noteworthy to mention that the prevalent intrinsic disordered regions in ORF2 protein could perform crucial regulatory functions by interacting with the other viral and host components.

Further, studies have shown the importance of MoRFs in several viruses [55, 56, 75–77]. The MoRF is defined as short segment within disordered regions of a protein (exists as IDPR or IDP) that upon binding with its partner undergoes a transition from disorder-to-order state [33]. These are segments that are prone to interactions [33]. Our study predicted MoRFs in ORF2 proteins by set of three predictors (DISOPRED3, IUPred3, and IUPred2A). DISOPRED3 server predicts the protein binding disordered regions within given target sequences [66]. We used DISOPRED3 for identifying IDRs as it provides significantly improved results over DISOPRED2 [66]. Additionally, IUPred members (IUPred3 and IUPred2A) were also utilized to predict the disordered binding regions within ORF2 protein sequences [78, 79]. IUPred3 and IUPred2A are webservers that allow identification of both disordered protein regions (using IUPred2/IUPred3) and disordered binding regions (using ANCHOR2) respectively [78, 79]. The identified multiple MoRFs at N-terminus of ORF2 protein further provided compelling evidence that ORF2 show propensity towards interaction with multiple partners with its N-terminus through its order/disorder tendency. According to some reports, ORF2 also contributes to interferon production and immunity recognition [80]. Thus, altogether these findings substantiate our results that the disordered N-terminal of the ORF2 protein interacts with various partners. Further, the ORF2 has also been linked to host tropism [81, 82]. This suggested the involvement of ORF2 in regulation and pathogenesis of HEV which shows consistency with recent report [54].

Further, studies have documented the role of post-translational modifications (PTMs) in various processes, such as folding of proteins, transduction of signals, regulation of transcription, progression of cell cycle, survival, and apoptosis [83]. Also, phosphorylation is essential for the establishment productive infection cycle for majority of intracellular pathogens [84, 85]. In RNA viruses, such as Alphaviruses [86, 87] and Flaviviruses [88–90], literatures have shown the essentiality of phosphorylation in critical protein functions. In this regard, we evaluated the phosphorylation scores using the DEPP online tool by analyzing the ORF2 sequences. Our phosphorylation patterns of ORF2 protein revealed that all the sequences consisted of P-sites. The observations revealed that P-sites were prevalent within disordered segments of the ORF2 polypeptide chains. This inferred strong correlation between protein phosphorylation and intrinsically disordered ORF2 regions. Thus, our findings are in agreement with previous report that has shown the interconnection between phosphorylated residues and overall disorderness in the proteins [91, 92]. It has been suggested that the disordered segment of protein regions displays sites for PTM is perhaps due to the conformational flexibility of display sites provided by the disordered regions in the proteins [93, 94]. Report has revealed that serines’s hydroxyl group act as target for phosphorylation by protein kinases, within the disordered protein segments [95]. Therefore, the higher predicted serine (phosphorylated) residue in ORF2 proteins revealed its interacting ability and flexible nature, ultimately relating its important role to protein regulation and its linked various biological processes.

Furthermore, the predicted molecular functions and biological processes (based on 3D ORF2 structured models) using COFACTOR algorithm [40, 41], such as, ion binding, metal ion binding, transition metal ion binding, ion-sulfur cluster binding, and protein binding, were predicted which clearly revealed the propensity of ORF2 to bind to a variety of molecules, such as ions, metals, and proteins. Such kind of interactive functions have been reported in regulation of various processes, like cellular signal transduction, phosphorylation, transcription, and translation [96]. Electron transport chain occurs inside mitochondria’s matrix and it involves redox reactions which are catalyzed by oxidoreductase enzymes. The predicted ETC and oxidoreductase activity suggested the ORF2 involvement in HEV regulation as mitochondrion serves as signaling hub for immune response [97, 98]. Also, a literature has evidenced the role of complex III (of electron transport chain) in HEV infection [99], which performs assorted biological functions [100, 101]. These predicted biological processes besides structural molecule activity clearly inferred the involvement of ORF2 protein in multiple crucial roles beyond its role as capsid protein [54]. These findings further substantiate our present hypothesis. On summing up our observations, it can be hypothesized that ORF2 is a protein with multiple functions and is involved in cell regulation and pathogenesis in addition to its role as a HEV capsid protein.

## Conclusions

The present study provides novel investigation on the biology of HEV ORF2 protein in terms of its disorderedness. This unique study employed disorder predictors to reveal peculiar intrinsic disorder patterns of HEV ORF2 that will help in understanding its behavior. The extent of intrinsic disorder distribution was calculated using different bioinformatics predictors by obtaining the ORF2 protein sequences from publicly available database. The initial comprehensive analysis of the ORF2 structured models showed significant percentages of coil. On analyzing the occurrence of intrinsic disorder extent, the ORF2 protein was revealed as IDP (highly disordered protein), thus suggesting its various significant roles in the life cycle of viruses. Further, the predicted MoRFs in the HEV ORF2 proteins suggested its propensity towards numerous disorder-based binding functions. These identified disordered regions in addition to disorder-based protein binding residues could perform diverse important roles such as viral replication, pathogenicity, and its particle assembly. Furthermore, the presence of several phosphorylation sites signified the involvement of ORF2 protein in various important mechanisms like cellular and signalling pathways. Moreover, structure-based prediction of crucial molecular functions and biological processes indicated multiple functions associated with it beyond its capsid function. Our study is further envisaged to provide critical information in studying the HEV ORF2 behavior.

## Data Availability

Not applicable.

## Abbreviations

HEV: Hepatitis E virus
GT: Genotype
ORF: Open reading frame
ORF2: Open reading frame 2
PONDR: Predictor of natural disordered regions
MoRFs: Molecular recognition features
IDRs: Intrinsically disordered regions
ORDP: Ordered protein
IDPR: Intrinsically disordered protein regions
IDP: Intrinsically disordered proteins
